# *Seek, and ye shall find*: Accessing the global epidemiological literature in different languages

**DOI:** 10.1186/1742-7622-5-21

**Published:** 2008-09-30

**Authors:** Isaac CH Fung

**Affiliations:** 1 Department of Infectious Diseases Epidemiology, Faculty of Medicine, Imperial College London, UK

## Abstract

The thematic series 'Beyond English: Accessing the global epidemiological literature' in *Emerging Themes in Epidemiology *highlights the wealth of epidemiological and public health literature in the major languages of the world, and the bibliographic databases through which they can be searched and accessed. This editorial suggests that all systematic reviews in epidemiology and public health should include literature published in the major languages of the world and that the use of regional and non-English bibliographic databases should become routine.

## Background

In 1976, Eugene Garfield, the founder-president of the Institute for Scientific Information (now Thomson Scientific [1]) sparked controversy in France with his article 'Is French Science too Provincial?' published in the leading French journal *La Recherche *[2]. By suggesting that 'French scientists must recognize that French is no longer *the *international language, and the adoption of English as the world language of science should be encouraged' (italics original) [3], Garfield drew strong and robust responses from French politicians and scientists [4]. In the ensuing years, the proportion of scientific articles indexed in international databases such as Medline [5,6] and the Science Citation Index [7] that is published in languages other than English has decreased. Medical journals published in French or German have declined in ranking based on impact factors [8]. By the late 1980s, Garfield noted that the 'French scientists seem to have implicitly acknowledged that English is, in fact, now the international language of science, if not in other areas of international activity' [9]. However, in a 2006 debate in the journal *PLoS Medicine*, Gerd Antes commented that in countries like Germany, most physicians and other health-care professionals are still unable or unwilling to read English in their daily routine [10].

Given that English has become the global language of science, non-English-speaking countries are at a disadvantage with respect to global scientific communication, and much is needed to remedy this situation. As highlighted in a recent comment in *New Scientist*, many local journals in the developing world are failing and yet their very existence is vital as a channel through which local research is publicised [11]. Unless non-English-speaking societies become genuinely bilingual, the 'overdominance' of the English language may impact adversely the work of epidemiologists and public health practitioners in non-English-speaking countries and, subsequently, their journal's publications [10,12].

## Global epidemiological literature in non-English languages and its retrieval

What is the current state of non-English epidemiological and public health journals? What are their prospects in the 21^st ^century? Has the growth of the internet and the movement towards 'Open Access' [13,14] made any difference to their survival? Are there any bibliographic databases that make these journals more readily accessible? What sort of epidemiological resources are available in these languages?

To address these questions, authors from diverse linguistic and geographical backgrounds have been invited to contribute to this thematic series on 'Beyond English: Accessing the global epidemiological literature'.

This series begins with Fung [15] providing a guide to Chinese-language biomedical journals, with an emphasis on those of most interest to epidemiologists. Highlighted is a variety of literature and the bibliographic databases through which it can be accessed. The wealth of the Chinese scientific literature is further illustrated by Liu and colleagues [16], using schistosomiasis research and control as an example. Barreto and Barata [17] highlight the significance of Portuguese-language literature and discuss the databases and portals, such as LILACS and SciELO, that are gateways to literature in Portuguese and Spanish. Williams and colleagues [18] review the historical development of public health research in the Spanish-speaking world and draw attention to the fast-growing research outputs from Latin America. Baussano and colleagues [19] present a comparative study of epidemiology and public health journals, databases and professional education in French, German and Italian, as well as the factors affecting their development in different linguistic, cultural and national contexts. Vlassov and Danishevskii [20] reflect on the history of the former Soviet Union and discuss why Russian biomedical journals and databases have much work ahead to compete with journals of the English-speaking world. Al-Shorbaji [21] describes the Index Medicus for the East Mediterranean Region, a gateway to the expanding literature of this locality. Lefebvre and colleagues [22] review the history of the Cochrane Central Register of Controlled Trials (CENTRAL) in the Cochrane Library and in particular how EMBASE contributed to this project. It is worth noting that EMBASE is rich in bibliographic records published in non-English languages that are not as readily available as those found in other popular sources such as PubMed. Lastly, Fung [23] comments on the current practice for citing non-English peer review publications among journals of relevance to epidemiologists.

## Systematic reviews should include literature published in the major languages of the world

It is crucial that all systematic reviews in epidemiology and public health aim to include literature published in the major languages of the world. While such a list is difficult to define, an example is the six official languages of the United Nations: Arabic, Chinese, English, French, Spanish and Russian [24]. However, selecting a canon of scientific languages, depends on the criteria on which it is based: one based on population size would promote those languages with a large number of native speakers, while one based on the volume of scientific literature published in the given language would reflect both traditional publishing practices and scientific outputs. With different criteria, languages such as Bengali, German, Hindi, Japanese, Portuguese, Swahili and Urdu would be included.

It must be stressed that the languages represented in this thematic series are by no means an exhaustive or authoritative list of what constitutes the major scientific languages of the world; indeed there are no South Asian or African languages discussed. Furthermore, one of the points of this thematic series is to highlight that the fact that the choice of language of publication for a given journal and the type of literature published within a given language are both context specific, so that for any given epidemiological question, every effort should be made to include a much broader list of non-English sources. The use of regional and non-English bibliographic databases, such as those in Spanish and Chinese, should become routine for performing literature searches. As international cooperation and collaboration in epidemiological and public health research has increased, it is now entirely feasible to enlist a team of authors with various linguistic abilities for a systematic review.

In today's global age of frequent international exchange and cross-cultural communication, increased access to non-English scientific literature will better serve epidemiology. *Emerging Themes in Epidemiology *welcomes the submission of papers that can be added to this thematic series in the future. It is hoped that this thematic series will encourage dialogue on a myriad under-utilised resources of epidemiological and public health data and, ultimately, facilitate the use of epidemiological data published in different languages.

## Abstracts in non-English languages

The abstract of this editorial has been translated into the following languages by the following translators (names in brackets):

• Chinese – simplified characters (The author) [see Additional file 1]

• Chinese – traditional characters (The author) [see Additional file 2]

• French (Mr. Philip Harding-Esch) [see Additional file 3]

• Spanish (Ms. Annick Bórquez) [see Additional file 4]

## Competing interests

The author declares that he has no competing interests. The author is one of the managing editors of the *Emerging Themes in Epidemiology*. He is the editor-in-charge for this thematic series.

## Authors' contributions

ICHF conceived the idea of this editorial, wrote it and read and approved its final manuscript.

## Funding

The author receives no funding for writing this editorial.

## Supplementary Material

Additional file 1 Abstract in Chinese (simplified characters). Click here for file

Additional file 2 Abstract in Chinese (traditional characters) Click here for file

Additional file 3 Abstract in French Click here for file

Additional file 4 Abstract in Spanish. Click here for file
