# Supplementary material for: Seek, and ye shall find: Accessing the global epidemiological literature in different languages
Source: Emerg Themes Epidemiol. 2008 Sep 30;5:21. doi: 10.1186/1742-7622-5-21 (PMC2570666; doi:10.1186/1742-7622-5-21)
Supplement: Additional file 1 — Abstract in Chinese (simplified characters). [file 1742-7622-5-21-S1.pdf]

Simplified Chinese / 简体中文

编者语

***寻则遇之*：获取不同语言的全球流行病学文献**

作者：冯俊熙 (Isaac Chun-Hai Fung)

摘要

《流行病学中的新近主题》的主题系列「英语之外：获取全球流行病学文献」强调在世界各大语言里，流行病学及公共卫生的文献均非常丰富，且都有文献数据库以供检索及存取。本编者语建议所有流行病学及公共卫生方面的系统综述，都应涵盖世界各大语言的相关文献，而使用地区性及非英语文献数据库去搜寻文献应成为工作常规。

（中文摘要由作者翻译）
