# Supplementary material for: Seek, and ye shall find: Accessing the global epidemiological literature in different languages
Source: Emerg Themes Epidemiol. 2008 Sep 30;5:21. doi: 10.1186/1742-7622-5-21 (PMC2570666; doi:10.1186/1742-7622-5-21)
Supplement: Additional file 2 — Abstract in Chinese (traditional characters) [file 1742-7622-5-21-S2.pdf]

Traditional Chinese / 繁體中文

編者語

尋則遇之：獲取不同語言的全球流行病學文獻

作者：馮儁熙 (Isaac Chun-Hai Fung)

摘要

《流行病學中的新近主題》的主題系列「英語之外：獲取全球流行病學文獻」強調在世界各大語言裡，流行病學及公共衛生的文獻均非常豐富，且都有文獻數據庫以供檢索及存取。本編者語建議所有流行病學及公共衛生方面的系統綜述，都應涵蓋世界各大語言的相關文獻，而使用地區性及非英語文獻數據庫去搜尋文獻應成爲工作常規。

（中文摘要由作者翻譯）
