# Supplementary material for: Seek, and ye shall find: Accessing the global epidemiological literature in different languages
Source: Emerg Themes Epidemiol. 2008 Sep 30;5:21. doi: 10.1186/1742-7622-5-21 (PMC2570666; doi:10.1186/1742-7622-5-21)
Supplement: Additional file 3 — Abstract in French [file 1742-7622-5-21-S3.pdf]

French / Français

Editorial

***Cherchez, et vous trouverez:* accéder à la littérature épidémiologique mondiale en différentes langues**

Auteur: Isaac Chun-Hai Fung

Résumé

La série thématique « Au-delà de l’anglais : accéder à la littérature épidémiologique du monde» du journal *Emerging Themes in Epidemiology* (*Thèmes émergents en épidémiologie*) met en évidence toute la richesse de la littérature concernant l’épidémiologie et les sciences de la santé publique existant dans les principales langues du monde, ainsi que les bases de données bibliographiques permettant de rechercher cette littérature et d’y accéder.

Cet éditorial propose que toute revue systématique dans les domaines de l’épidémiologie et des sciences de la santé publique inclue la littérature publiée dans les principales langues du monde, et que les bases de données régionales dans des langues autres que l’anglais soient consultées systématiquement.

Traduit de l’anglais par Philip Harding-Esch
