# Supplementary material for: Seek, and ye shall find: Accessing the global epidemiological literature in different languages
Source: Emerg Themes Epidemiol. 2008 Sep 30;5:21. doi: 10.1186/1742-7622-5-21 (PMC2570666; doi:10.1186/1742-7622-5-21)
Supplement: Additional file 4 — Abstract in Spanish. [file 1742-7622-5-21-S4.pdf]

Spanish / Español

Editorial

***El que busca encuentra*: Accesar la literatura global en epidemiología en distintos idiomas**

Autor: Isaac Chun-Hai Fung

Resumen

La serie temática de *Temas Emergentes en Epidemiología*: “Más allá del inglés: accesar la literatura global en epidemiología”, resalta la riqueza de la literatura en epidemiología y salud pública en los principales idiomas del mundo así como las bases de datos bibliográficas a través de las cuales se pueden buscar y accesar. Esta editorial sugiere que todas las revisiones sistemáticas de la literatura en epidemiología y salud pública deberían de incluir la literatura publicada en los principales idiomas del mundo y que el uso de bases de datos bibliográficas regionales y en otros idiomas que el inglés debería de convertirse en rutina.

Traducido al español por Annick Bórquez
